# 3D printing vs traditional workflow for the fabrication of mandibular implant overdentures: study protocol for a mixed-methods cross-over RCT

**DOI:** 10.1186/s13063-024-08097-7

**Published:** 2024-04-16

**Authors:** Dana Jafarpour, Nesma El-Amier, Jocelyne Feine, Christophe Bedos, Samer Abi-Nader, Shahrokh Esfandiari, Tibor Shuster, Elizabeth Zimmermann, Raphael de Souza

**Affiliations:** 1 Faculty of Dental Medicine and Oral Health Sciences, Strathcona Anatomy and Dentistry Building, McGill University, 3640 University Street, Room #M/65A, Montréal, QC H3A 2B2 Canada; 2 Faculty of Dentistry, Mansoura University, Mansoura, Egypt; 3 Faculty of Dental Medicine, Faculty of Medicine, Université de Montréal, Montreal, Canada; 4 Department of Family Medicine, McGill University, Montreal, Canada

**Keywords:** 3D printing, CAD/CAM, Mandibular overdenture, Costs and cost analysis, Cross-over studies, Dental care for aged, Edentulous mouth, Implant-supported dental prosthesis, Patient satisfaction, Removable prosthodontics

## Abstract

**Background:**

Complete tooth loss is a significant global oral health issue, particularly impacting older individuals with lower socioeconomic status. Computer-assisted technologies enhance oral healthcare access by the elderly. Despite promising in vitro reports on digital denture materials, evidence from randomized clinical trials (RCTs) is lacking to verify their performance. This cross-over RCT will investigate whether 3D-printed implant-retained mandibular overdentures (IMO) are more satisfactory for edentulous seniors than those made through traditional methods.

**Methods/design:**

We will recruit 26 completely edentulous participants (any sex/gender) based on the following eligibility criteria: age ≥ 60 years, no tooth extraction in the past 12 months, two implants in the lower jaw, and need for new dentures in both jaws. Each participant will receive two denture pairs, either manufactured by 3D printing or traditionally, to be worn in a random order. A timeline of 3 months with each denture pair will be considered for outcome assessment (total: 6 months). Patient satisfaction with dentures will be measured by the McGill Denture Satisfaction Questionnaire. We will evaluate other patient-reported outcomes (including oral health-related quality of life) as well as clinician-assessed quality and cost. At the end of the trial, participants will choose which denture pair they wish to keep and interviewed about their experiences with the 3D-printed IMO. The quantitative and qualitative data will be incorporated through an explanatory mixed-methods strategy. A final quantitative assessment will happen after 12 months with the preferred IMO to assess the long-term performance and maintenance needs.

**Discussion:**

This mixed-methods RCT will explore patient experiences with 3D-printed IMOs, aiming to assess the potential for altering clinical practice and dental public health policies. Our results will inform policies by showing whether 3D printing offers comparable outcomes at lower costs, facilitating greater access to oral care for the elderly.

**Trial registration:**

ClinicalTrials.gov, NCT06155630, Registered on 04 December 2023. https://classic.clinicaltrials.gov/ct2/show/NCT06155630

**Supplementary Information:**

The online version contains supplementary material available at 10.1186/s13063-024-08097-7.

## Background

Complete tooth loss or edentulism remains one of the most burdensome oral health issues globally. Its prevalence is clustered in elderly populations and tends to remain high for many decades [1]. Edentulism can devastate well-being [2], with depressive symptoms worsening after complete tooth loss [3]. Poorer oral function after tooth loss, including impaired mastication [4], is closely linked to psychosocial discomfort and lower self-esteem [5, 6]. Moreover, edentulism has been shown to have likely systemic implications, as evidenced by its association with disability and earlier mortality in the elderly [7].

Even if implant-retained overdentures restore oral functionality and improve nutrition for the elderly [8–10], significant obstacles limit access to such care: (a) public funding for adult oral healthcare is limited, generally excluding prosthetic treatments for most without teeth [11, 12]; (b) high costs of private care, often not reimbursed, disproportionately affect those in lower socioeconomic groups, restricting their access to necessary dental services [13, 14]; (c) the need for multiple clinical visits, sometimes over six, poses challenges in terms of mobility and costs, particularly for those in long-term care or during health crises like pandemics [15, 16].

Computer-assisted technologies can greatly improve access to oral healthcare by the elderly. Fewer appointments for denture treatment, i.e., two to four, instead of five as with the conventional techniques, can reduce patient costs [17]. Patients can receive 3D images of their face with future dentures by internet, thus avoiding a clinical visit [18]. Moreover, digital files can be used to remake the same dentures in the absence of the patient, whereas analogic techniques need a repetition of the original workflow [19].

Among the computer-aided designed and manufactured (CAD/CAM) options for denture fabrication, 3D printing (or additive manufacturing) stands out as a highly promising technology. In comparison to traditional and other CAD/CAM workflows, 3D printed dentures have the potential to minimize material waste while achieving high speed and quality [20]. Even with promising in vitro reports of digital denture materials [21–24], however, evidence from randomized clinical trials (RCTs) is still missing to verify their clinical performance. Our recent scoping review (search update: March 1, 2023) [25] found no RCT on 3D-printed implant-retained mandibular overdentures (IMO), which have been considered the standard of care for complete edentulism by international consensuses [26, 27].

In addition, treatment success with dentures mostly depends on positive patient experiences. Integrating patient attitudes into the final dentures is vital for favorable results. Thus, patient-reported outcome measures (PROMs) are the core criteria in evaluating denture care, as for many healthcare interventions [23]. Yet, no study has explored patients’ experiences with the CAD/CAM dentures. In other words, it is unclear how patients perceive CAD/CAM dentures and whether they have a satisfactory performance from patients’ perspectives.

### Objectives and hypothesis

We aim to conduct the first mixed-methods cross-over RCT to determine the efficacy and patients’ experience with 3D-printed implant-retained mandibular overdentures (IMO), compared to the traditional method, for independently living edentulous seniors who have two implants in the anterior mandible, as purported by the McGill Consensus on Implant Overdentures [26]. Our null hypothesis is that IMOs produced by computer-aided design and 3D printing are as satisfactory to edentulous seniors as those fabricated using traditional methods.

## Methods

### Design and setting

**Fig. 1:**
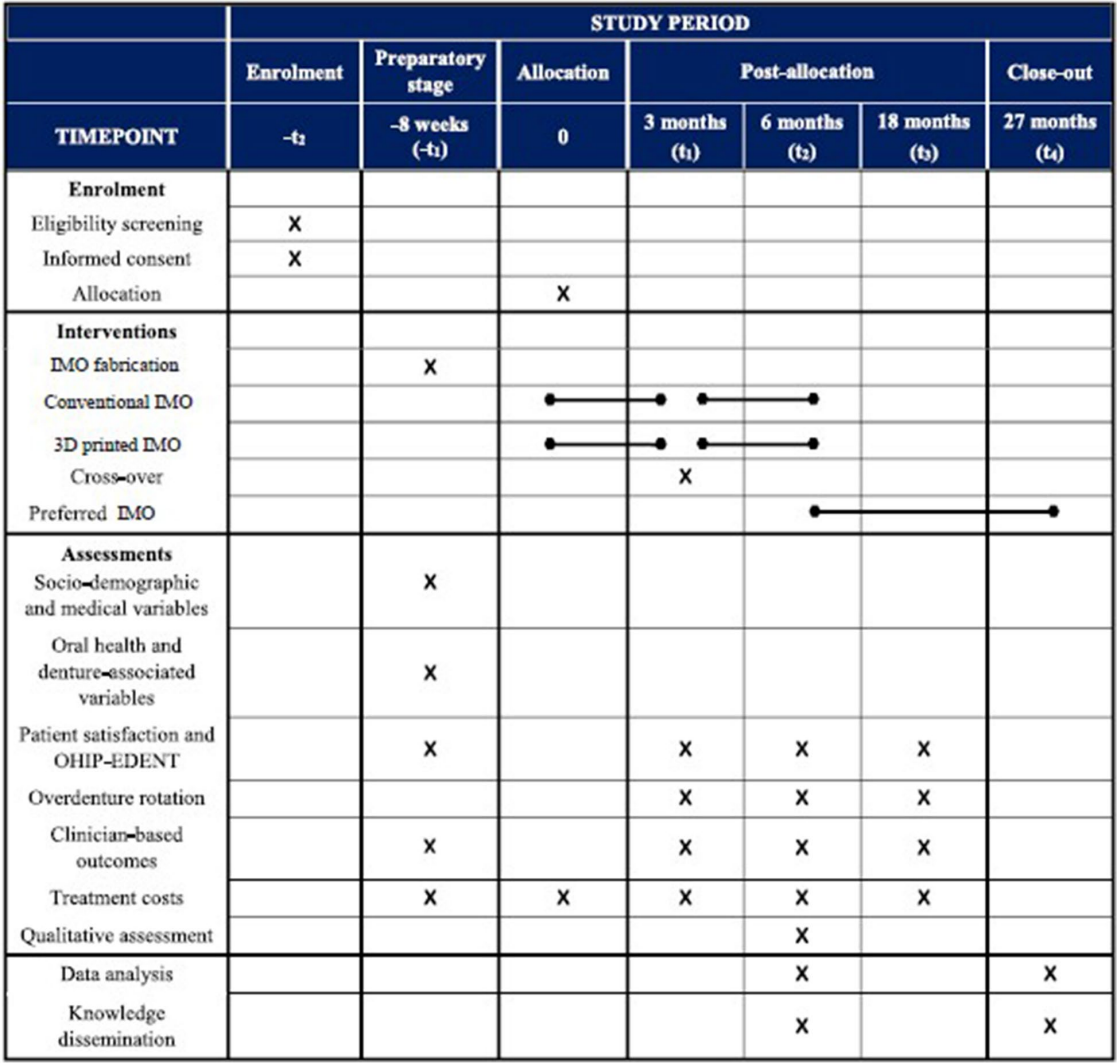
The study protocol based on Standard Protocol Items: Recommendations for Interventional Trials (SPIRIT)

This mixed-methods cross-over RCT will compare one experimental intervention (CAD/CAM dental prostheses) versus an active comparator (conventional denture fabrication methods). Outcome assessment will take place 3 months after each intervention, up to a total follow-up of 6 months. This will be a single-center RCT, conducted at the Faculty of Dental Medicine and Oral Health Sciences, McGill University (Montreal, Canada). The creation of this report adhered to the guidelines outlined in the Standard Protocol Items: Recommendations for Interventional Trials (SPIRIT) [28]. Figure 1 illustrates the standard protocol items diagram as suggested by SPIRIT, while the SPIRIT checklist for this study is provided in Additional file 1.

### Eligibility criteria

This trial will recruit edentulous patients seeking treatment with IMOs and maxillary complete dentures at McGill University.

The inclusion criteria is as follows: (i) elderly according to the age cut-off purported by the World Health Organization (age ≥ 60 years) [29] and living independently; (ii) completely edentulous; (iii) no tooth extraction within the past 12 months; (iv) two implants symmetrically distributed in the anterior mandible for three or more months before the trial interventions; (v) desire to receive both upper denture and lower IMO with new stud attachments; (vi) good understanding of spoken and written English or French; (vii) ability to provide written informed consent.

The exclusion criteria are as follows: (i) severe systemic disease or needing frequent hospitalization (i.e., American Society of Anesthesiologists Physical Status class > II) [30]; (ii) evidence of cognitive or motor impairment; (iii) acute or chronic symptoms of parafunctional or temporomandibular disorders; (iv) intraoral pathologies, either acute, progressive, potentially malignant, or capable to hamper denture fit; (v) signs of endosseous lesions or residual dental structures in panoramic radiographs; (vi) signs of implant failure, including clinical mobility, peri-implant radiolucency, unacceptable bone loss (0.2 mm/year after 1st year, or < 2 mm any time) and/or persistent signs/symptoms of pain, neuropathy, infection, and/or exudate [31].

### Participant recruitment

We will recruit participants from the Greater Montreal area by verifying lists of prospective and past patients in our faculty. This will be done directly with the faculty’s clinical staff to reach those patients. Priority will be given to patients treated in recent years who may need to have their old IMOs replaced, e.g., 63 patients from 2010 to 2018 (around eight patients/year). Staff involved in clinical services will mention this study to patients and, if the latter have interest, refer them to our trial coordinator (TC). Patients without implants will be included if they comply with inclusion criteria #4 (two implants in the lower jaw) within the recruitment period.

Recruitment rate will be based on the ability of the researchers to provide treatment rather than participant availability, which is set at three patients/month. Recruiting 37% of the 63 patients available from 2010 to 2018 would reach the planned sample size (*n* = 26). Participants will be assigned to interventions with the recruitment flow, which will take nine months (from the 5th to the 14th month). Recruitment and care provision to participants will be spread over a 9-month period to reduce seasonal variation in their responses.

### Trial interventions

**Fig. 2:**
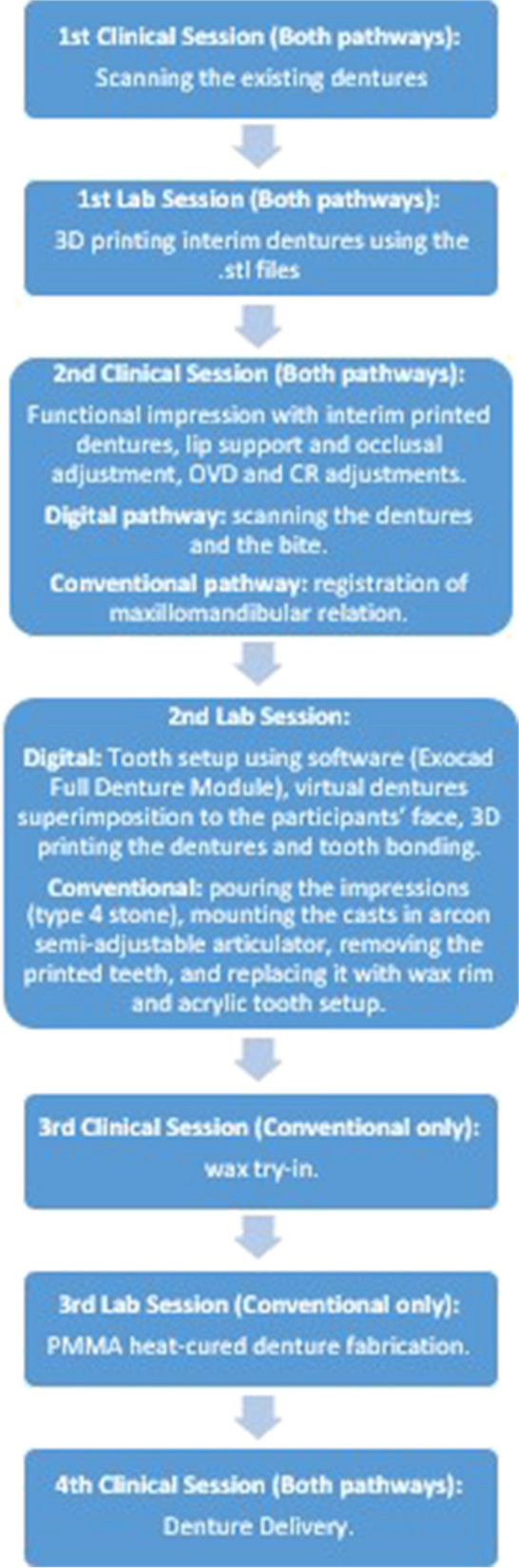
The trial intervention procedures

After screening and obtaining patient consent, participants will return to start denture fabrication (Fig. 2). Both denture pairs will be fabricated simultaneously and delivered in random order. Existing attachments will be replaced by Novaloc abutments of adequate cuff height (external margins 1 mm above the mucosa) and yellow/medium retentive matrices.

Participants will come for a first appointment for scanning their existing dentures, with new abutments (manual torque) and attachment housings in place. The first visit will be the same for both conventional and digital pathways and will consist of scanning the patients’ existing upper complete denture and IMO. The existing dentures will be scanned by using an intraoral scanner (Cerec Primescan, Dentsply Sirona, Charlotte, NC, USA). In the lab, the resulting.stl files will be 3D printed using a Max DLP 3D printer (Asiga, Alexandria, Australia).

In a second clinical appointment, the interim printed dentures will be adjusted with wax for the desired lip support, occlusal plane, occlusal vertical dimension (OVD), and centric relation (CR). Secondary impressions (regular PVS – Examix, GC America, Alsip, IL, USA) will be taken under the intaglio to refine fit, with the new abutments and housings in place. Maxillary and mandibular interim printed dentures will be scanned separately and in occlusion. The face of the patients will also be scanned by a face scanner (Shining 3D face scanner, Shining 3D Ltd.).

In the second lab session,.stl files will be superimposed for the digital pathway. Another software (Exocad Full Denture Module, Exocad GmbH, Darmstadt, Germany) will be used to set teeth and denture bases virtually; virtual dentures will be superimposed to the facial images to estimate final results.

Denture bases will be manufactured by a Max DLP 3D printer with Dentca Denture Base Resin (Dentca Inc., Torrance, USA) at 100 μm/layer and supports on the flanges’ facial surface. A 0.3-mm relief will be applied in the sockets dedicated to receive teeth and attachment housings. Following washing in isopropyl alcohol (5 min), dentures will receive Portrait 3D teeth (Dentsply Sirona, Charlotte, USA), treated by the Fuse 3D Denture Bonding System (Dentsply Sirona). Uncured base resin will be used to lute teeth and cured under UV. Final polymerization will take place in a dental polymerization equipment (Flash, Asiga) for 30 min. Dentures will be finished and polished, and sandblasted (50 μm aluminum oxide) in the attachment sockets.

For the conventional pathway, the second lab session will consist of pouring the impressions (type IV stone), mounting the casts in an Arcon semi-adjustable articulator, removing the printed teeth, and replacing them with wax rim and acrylic tooth setup (Portrait, Dentsply Sirona – same shape, size, and shade used for 3D-printed dentures).

A third appointment will be used for wax try-in for the conventional pathway. For the digital pathway, participants will have a chance to appraise their smile on a computer screen (virtual try-in, done remotely) and request modifications.

Denture bases will be manufactured with conventional heat-polymerized resins, and participants will return for a fourth appointment for delivery, including chairside pick-up of attachments (GC Reline resin; GC America Inc., Alsip, USA). Two short-term adjustments will be scheduled 24–72 h and 7 days after delivery, and then weekly until the dentures are comfortable.

In the fourth appointment, the following will be done for the digital pathway: denture delivery, including abutment insertion (torque: 35 Ncm), chairside attachment pick-up, and first adjustments. Chairside pick-up will use a hard reline resin (GC Reline), to be handled as per manufacturer recommendations.

For both interventions, we expect to adjust most dentures at two post-delivery appointments. In a previous RCT on conventional complete dentures, 10% needed more than four appointments, with a maximum of six appointments for a single participant (total *n* = 39) [16].

Participants will be scheduled for outcome assessment at 3 months following the delivery of each denture pair. A 3-month period is enough to elicit stable patient perception of existing dentures but will not induce significant wear/degradation of dental biomaterials or poor fit due to changes in intraoral tissues. Moreover, extending each follow-up from 3 to 6 months is not expected to result in changes of ratings of patient satisfaction with received dentures, even if denture adjustments are part of the 3-month period [32, 33].

### Participant allocation and minimization of bias

Assignment to either experimental or comparator as the first method will take place immediately after delivery adjustments of both denture pairs. The sequence for the interventions will be decided at the individual level following a list of random numbers (1:1 ratio), according to permuted blocks of varying sizes. Randomization will be performed by the trial coordinator (TC; uninvolved with clinical and follow-up procedures) and will take place at the individual level. The TC will retain a list of fabrication methods per denture pair and identify them as #1 and #2, depending on which will be used first. Random allocation to each intervention sequence will be concealed until both denture pairs are ready for delivery.

The TC will be the only person with access to the randomization codes. It is known that gender influences patient perceptions of received dentures, e.g., elderly women tend to give a higher value to esthetics than their male counterparts [34, 35]. Therefore, the sample will be stratified based on male/female, to analyze possible effects of gender on trial outcomes.

Regarding blinding, participants will be unaware of received interventions, and a researcher uninvolved in the clinical procedures will collect outcome data. Blinding will be lifted only after data collection is complete. Since digital manufacturing may have some specific features (like a subtle staircase topography), blinding effectiveness will be verified at each 3-month follow-up. Participants will answer which denture pair is in use, and to grade their conviction of their response from 0 to 10 (“not at all certain” and “extremely certain,” respectively) [36].

### Outcome measures

The primary outcome of this trial will be the general satisfaction of participants with their full dentures, in line with its ultimate goal. In addition, secondary measures will include satisfaction-specific aspects (e.g., chewing ability and esthetics), oral health related quality of life (OHQoL), clinical quality of the dentures, and treatment costs, all based on a public health system perspective.

1. The McGill Denture Satisfaction Questionnaire (MDSQ) [37, 38] will be used to measure overall satisfaction, and satisfaction with specific aspects of the denture—ability to chew, comfort, stability, esthetics (appearance), ability to speak, and ability to clean. Participants will rate their satisfaction on a 100-mm visual analogue scales (VAS) with anchors representing “no satisfaction at all” to “complete satisfaction.” Participants will receive training with VAS before answering the MDSQ. Previous studies have shown good properties for the MDSQ. Besides good internal consistency and reproducibility [38, 39], its ability to discriminate between different clinical conditions denotes good construct validity [38, 40, 41]. At the last follow-up, participants will be asked about which denture pair they prefer, if any, and their reasons.
2. OHQoL: This construct, conceptualized as the “subjective evaluation of the individual’s oral health, functional well-being, emotional well-being, expectations and satisfaction with care, and sense of self” [42], will be assessed by the Oral Health Impact Profile for Edentulous Patients (OHIP-EDENT) questionnaire. OHIP-EDENT is a short version (20 questions) of the original 49-question OHIP (Oral Health Impact Profile) tested specifically with edentulous individuals [43]. Questions can be grouped into subscales, corresponding to domains/dimensions of perceived impact, including functional limitation or social disability. This short version shows good reliability and discriminant validity, akin to the original OHIP [43]. Despite the seven original subscales, a four-domain model based on recent factor analysis studies will be used [44, 45].
3. Clinical denture quality: This trial will use the Functional Assessment of Dentures (FAD) instrument to assess denture quality [46, 47]. FAD is composed of questions about relevant clinical parameters, including dental occlusion/articulation, denture retention, and stability. A single dentist will apply the instrument without removing the dentures from the mouth, which will ensure that s/he cannot see the staircase topography of CAD/CAM fabrication. The color of the denture base materials will be verified at baseline and after 3 months with a Vita EasyShade portable spectrophotometer [48]. Upper dentures will be placed on a black background and three measures will be taken from the center of the palatal vault, at the polished side. Color measures will be expressed according to the CIELCh and CIELab systems. Clinically evident damage (e.g., fractured base or teeth, stains) to denture constituents will be also reported.
4. Cost: Data on both the direct and indirect costs of each fabrication method will be gathered, as done previously [16, 49]. The number of clinical visits for denture fabrication and adjustments will be reported separately, including non-scheduled visits. Total cost per fabrication method will be described in terms of expenses with human resources (time and CAD$) and materials (consumables/equipment use, CAD$). Yearly cost differences, or $(Δ), will be calculated based on the expected lifetime of 5 years for a pair of dentures, by dividing total cost differences by five. After the last follow-up, participants will answer four questions on their willingness-to-pay for each denture pair: (1) If you were to choose A over B, how much are you willing to pay for it? (2) Are you willing to pay the $(Δ) to have A over B? (3) Are you willing to pay the $(Δ) to have A over B by monthly installments (12 months)? (4) Do you think that a public health plan should cover the cost of these treatments? Both or only A or B.

The cost-effectiveness of both methods will be compared by using overall patient satisfaction to measure the effect of interventions. Economic analysis will have the perspective of the public health system of Quebec. All expenditures and resources through all stages of dental care (from clinical exam to denture adjustments) will be included, considering a short-term/3-month time frame. Results will be expressed by the incremental cost-effectiveness ratio (ICER).

5. Adverse effects: All adverse events during the RCT will be recorded at each post-delivery appointment and follow-up. Common events, i.e., mucosal injuries and difficulties with new dentures, will be rated on a three-point ordinal scale [50]. More uncommon (nausea, change in taste and lingering speech difficulties) and rare events (allergy to denture materials) will be reported on a binary scale.
6. Choice of overdenture and patient experiences: A qualitative analysis will be carried out to better understand patients’ perception of CAD/CAM dentures and to find out any emergent themes besides (1) patients’ reasons for choosing a specific denture pair and (2) their experience of using a 3D-printed IMO. By adopting a descriptive approach, individual semi-structured interviews will be carried out to obtain an in-depth understanding of patients’ experiences and preferences [51].

### Outcome assessment timeline

1. Screening: The RA will invite potential participants, answer questions, and gather informed consent. The RA will complete a screening form checking eligibility criteria for all approached individuals. Panoramic radiographs will be requested, and recruitment will be conditional based on the results from the imaging (as in the exclusion criteria). As a piloting process, we collect data on gender conformity by two questionnaires, the Conformity to Feminine Norms Inventory-45 and Conformity to Masculine Norms Inventory-30 [52, 53].
2. Baseline: Participants will return for baseline evaluation, *intraoral scanning*, and collection of sociodemographic information (including gender questionnaires) and medical and dental history (including patient experience with previous dentures). Participants will be asked to complete the MDSQ and OHIP-EDENT, and a dentist with experience in providing full dentures will apply the FAD instrument.
3. Delivery and denture adjustments: Immediately after the end of the delivery appointments, the FAD instrument will be applied for each denture pair (same dentist as on baseline). The adverse effects form will be completed at the same time, as well as during each post-delivery appointment, scheduled or not.
4. First follow-up (3 months): Participants will complete the outcome data questionnaires, the MDSQ and OHIP-EDENT. We will administer both questionnaires with a tablet computer away from the clinical setting. In turn, we will complete the FAD and adverse effects form, as described in the previous paragraph plus the colorimetric evaluation of denture bases.
5. Second follow-up (6 months): Same as in previous time point (questionnaires and colorimetric analysis). At the end of the trial, participants will choose which overdenture they wish to keep. When faced with a choice, one is presented with the choices and the associated costs, which allows for a rationale decision. This is particularly important as dentures are typically not covered by the health care provider. Then, we will conduct individual semi-structured interviews about their experiences with the IMOs. The rich descriptive data obtained by qualitative interviews will help further explicate the quantitative findings. An experienced qualitative researcher will be responsible to conduct the interviews outside of the clinic using an interview guide with open-ended questions. Given the explanatory nature of this mixed-methods design, the interview guide will be created iteratively based on the quantitative outcomes which require further explanation. Each interview will be audio-recorded and transcribed verbatim.

At baseline, we will register time (professional and patient) and materials use for cost analyses. The same procedures will be repeated during every appointment in the trial, scheduled or not.

6. Long-term follow-up: Once participants provide 6-month data and choose the denture pair they wish to keep, we will continue their follow-up. For those participants who have no preference for one specific pair, the last pair used will be kept. Then, we will schedule them for yearly appointments until 5 years, during which the same outcome data will be gathered. In the meantime, participants may contact the research team for unscheduled appointments. Any event such as maintenance and clinical complications, as well as time and procedures done, will be reported as part of collected outcome data.

### Sample size estimation

The planned enrollment comprises 26 participants, based on overall patient satisfaction. A minimal important difference of 10 mm (10% of the VAS) was used for the estimation, as done in previous RCTs [41, 54]. A standard deviation of 7.5 mm was chosen for the difference in satisfaction [55]. Considering a 2-sided alpha of 0.01 to compensate for the number of secondary outcomes and a power of 90%, the RCT requires *n* = 21 for superiority hypothesis testing (i.e., the confidence interval for between-treatment differences would exclude zero) [56]. The final sample size is drawn from including further 20% to the planned *n* to compensate for possible dropouts; although withdrawals will unlikely pass 10% [41, 55, 57], additional participants may be lost due to aging-related issues (e.g., worsening of systemic diseases, and death).

### Adherence to protocol and losses to follow-up

Previous studies by our group reveal high study adherence rates, with > 90% wearing their full dentures over the course of 6 months [41, 57–59]. This RCT provides variations of a treatment sought by participants at university clinics, with no major change in their routine. Subsequent follow-ups following the end of this RCT will also elucidate adherence rates for longer periods. As much as possible, we set up the intervention schedule, follow-up, and data collection to resemble traditional oral healthcare procedures. We will also have the RA communicate with the participants using their preferred communication method (e.g., phone, text, e-mail) to verify possible adverse events, such as pain under the denture base, that may require a rapid adjustment visit. Those events and consequent conducts will be reported as part of the outcome variable “5. Adverse effects”.

### Analytical plan

#### Quantitative analysis

We will enter and analyze outcome data in a blind fashion at the end of data collection. The TC will enter data in spreadsheets by randomly coding the interventions as 1 and 2, and the data analyst will be unaware of their meaning until the end of statistical testing. Data will undergo descriptive analysis, with substantial deviations from normality leading to variable transformation. Generalized estimating equations (GEE) will be used to test the effect of interventions, follow-up time, and gender as independent variables, with 95% confidence intervals. Other approaches to assess the primary outcome will involve the inclusion of age, baseline results, and previous denture wearing in separate models, one covariate at a time.

A limitation with regard to GEE is that compared to parametric normal theory methods that necessitate the missing data to be missing at random, GEE methods impose a more stringent condition, requiring the data to be missing completely at random. Our results will be evaluated according to the intention-to-treat principle. In the case of unbalanced missing data among interventions or loss of 5% (*n* > 2) or more of participants, different strategies will be attempted for imputing primary outcome data, as recommended by Dziura et al. [60]; multiple imputation will be used for patient satisfaction based on least squares regression with at least five datasets. In the case of “missing not at random” (MNAR) data, analyses will be repeated after a baseline-observation-carried-forward approach (i.e., withdrawn participants will be considered as dissatisfied as prior to receiving dentures). A second analysis will be performed with imputed values and cross-checked.

#### Qualitative analysis

Once the interviews are completed and transcribed, we will use the MaxQDA software for thematic analysis. Transcripts will be cut into meaningful segments and coded. Analysis will initiate by a deductive coding strategy based on the interview questions and the theoretical framework of denture satisfaction [37, 38]. Then, we will proceed by adopting an inductive analysis to add any emerging codes. By an iterative process, the coded segments will be regrouped into relevant themes linked to our study objectives [61, 62]. Methodological rigor will be warranted in the study, including member checking before or during data analysis, to meet trustworthiness, credibility, and transferability [63], that is, the results of the study will be formally or informally discussed with participants. To enhance coding quality, the qualitative researcher and one of the team members will independently code two transcripts chosen at random and then meet to compare/revise the codes if needed. Lastly, the qualitative data will be integrated into quantitative data by an explanatory strategy.

### Data management, monitoring, and auditing

Two independent researchers will regularly review the collected data as part of a data monitoring committee. Additionally, McGill University’s Research Ethics Board (REB) Office retains the authority to conduct an independent audit at any point in time.

### Risks, participant safety, and trial adherence

This study represents minimal safety risk for participants, since all procedures are comparable with nonsurgical oral healthcare (i.e., clinical exam, intraoral molding). The number of appointments to fabricate and adjust provided dentures is similar to what is done in standard practice, and all materials to be used are approved for patient use by Health Canada. Potential participants will receive a complete explanation of the RCT, including potential risks before invitation to sign the informed consent.

All denture materials are licensed for patient use and sold in Canada and United States. This way, risks associated with treatment are the same expected for minor oral surgery and standard dental implants/dentures. Participants may experience sore spots under the dentures after the placement of retentive components. If this happens, the dentures will be adjusted as necessary. Allergic reactions to dental materials (such as the acrylic mixture used to bond components and denture) are rare but might also occur. We do not expect risks or complications from the x-rays or other exams. This includes data collection and interviews.

We will monitor the participants for the duration of the research appointments. If lower denture breakage happens after installing retentive components during the study timeline, we will fix/repair it at no cost. Any dental treatment need will be managed by our research team or referred to professionals outside our research team. The latter case may arise, for example, if a participant requests more implants to retain their dentures.

### Confidentiality

Each participant’s quantitative and qualitative data will be assigned an identification code to ensure confidentiality. The information connecting participants’ identities to the codes will be securely stored in a password-protected file and computer. The final research forms, x-rays, and collected data will be sent to the primary investigator’s office and stored for 25 years for the exclusive objectives of this study and then destroyed. These will be kept secure by a password to which only the principal doctor will have access.

### Dissemination and knowledge transfer

Knowledge translation will target (i) the scientific community and (ii) the public health and professional sectors. For the first, the team will publish reports in high-impact dental journals, as done with our previous clinical studies [41, 58]. The group will present results in scientific conferences aiming at dental professionals and researchers, as well as industry professionals, including the General Session of the International Association for Dental Research (IADR). For the second, our team will describe clinical and laboratory procedures as appendices of our scientific papers, besides a short book and YouTube videos, both directed to clinicians. The results of this study will be used to develop continuing education courses and webinars that explain the use of digital dentures based on our experiences and research findings. In addition, we will produce patient education materials, including a brochure and a video that describe the potential benefits and limitations of CAD/CAM technology for denture fabrication.

## Discussion

Despite promising results, there is a scarcity of RCTs comparing CAD/CAM to conventional full denture fabrication methods, either implant-retained or conventional [25]. As of March 2018, only two clinical studies have compared full dentures fabricated by CAD/CAM and conventional methods [64]. Both of these studies were non-randomized, with one based on treatment provided by dental students [65] and the other considering surrogate measures [66]. A recent systematic review (updated: October 2019) confirms that CAD/CAM technologies produce dentures that fit intraoral tissues better than conventional methods [67]. That review was restricted to in vitro studies, however. To appraise the state of the current literature regarding digital removable dentures, we re-ran the electronic search strategies of those reviews and found no cross-over clinical trial comparing CAD/CAM to conventional implant-retained dentures. Among those newly published comparative studies on CAD/CAM conventional dentures, two were prospective clinical studies [65, 68], two were retrospective studies [69, 70], one was cross-sectional [71] (all five conducted in student clinics), and one was a non-randomized trial [72], besides one RCT on conventional dentures made with a closed workflow (Dentca) [73]. Besides, no study has so far explored patient experiences with the CAD/CAM dentures. Given the impact of dental prostheses on the oral health of edentulous patients, it is essential to document the clinical and patient-reported performance of digitally fabricated dentures.

Determining the potential benefits of CAD/CAM full dentures for seniors demands high-quality comparative evidence (i.e., RCT). This proposal is the first step to determine the advantages and limitations of that novel technology in treating the elderly with implant overdentures. Utilizing qualitative methods allows for a comprehensive exploration of patients’ experiences, as evidenced by a previous study conducted by our team, which examined the reasons for declining treatment with implant overdentures [38]. Employing a mixed-methods approach will integrate the quantitative findings from the clinical trial by offering an in-depth interpretation of patient perspectives through qualitative methods [58].

Anticipated outcomes are expected to hold significant relevance for clinicians administering implant-assisted treatment to edentate patients. The resulting guidelines and recommendations have the potential to enhance dental prosthetic care for the edentate elderly population, a substantial portion of whom face challenges accessing more intricate treatment modalities.

Results will be important to guide public health systems and practitioners in the adoption of CAD/CAM to streamline denture provision. Better outcomes will enhance the potential access to care by edentulous seniors; government-subsidized programs, such as the recently launched Canadian Dental Care Plan for seniors, will be able to provide IMOs with lower costs and/or fewer restrictions. Less appointments and better infection control will also reduce risks for elders to contract diseases such as COVID-19 and flu-like infections in the dental setting. Possible remote care (virtual try-in of new dentures, ordering remakes at distance) also reduces infection risk. Better knowledge of patient perceptions of CAD/CAM IMOs will highlight the potential barriers and opportunities for using digital prostheses in dental practice. It will also help understand if the CAD/CAM technology has reached its goal of offering a superior/cost-effective treatment.

## Trial status

Recruiting since January 2024.

### Supplementary Information

**Additional file 1. **SPIRIT Checklist.

**Additional file 2. **Funding documentation.

**Additional file 3. **Ethical approval document.

**Additional file 4. **Consent form in English.

## Data Availability

For depositing research data, we might deposit anonymized datasets in the McGill Dataverse repository upon request. According to McGill, all data are stored securely on servers located in Canada. The anonymized data may be published or shared during scientific meetings; however, it will not be possible to identify the participants.
