# Supplementary material for: 3D printing vs traditional workflow for the fabrication of mandibular implant overdentures: study protocol for a mixed-methods cross-over RCT
Source: Trials. 2024 Apr 16;25:267. doi: 10.1186/s13063-024-08097-7 (PMC11022432; doi:10.1186/s13063-024-08097-7)
Supplement: Supplementary file 2 — Additional file 2. Funding documentation. [file 13063_2024_8097_MOESM2_ESM.pdf]

International Team for Implantology

Dr Raphael de Souza
McGill University
Faculty of Dental Medicine and Oral Health Sciences
3640, University St. # M/65A
Montreal H3A2B2
CA
raphael.desouza@mcgill.ca

Basel, 25 May 2023

Grant confirmation letter

To whom it may concern

With reference to your grant application submitted to the ITI, we take pleasure in confirming that the ITI Research Committee has agreed to support project "3D printing vs traditional workflow for the fabrication of mandibular implant overdentures: A randomized crossover clinical trial" to be led by Dr. de Souza with an ITI research grant to the amount of USD 139.734,00. This grant applies to the project as approved by the ITI Research Committee. Any changes to the protocol or design throughout the duration of the project must be communicated to ITI Headquarters in advance.

The research project begins in 01/08/2023 and the projected end date is 31/07/2026.

The grant will be transferred to the bank named below to an account whose details were communicated to ITI Headquarters as follows:

Name of the bank:	RBC Royal Bank
Address of the bank:	Montreal Main Branch, 1Place Ville Marie, Montreal, QC H3C 3B5
Account holder:	The Royal Institution for the Advancement of Learning McGill University

The grant is to cover expenses associated with the project above. The payment schedule is as follows:

1.	After provision of ethical approval (August 2023):	USD 70.000,00
2.	After provision of status report 1 (August 2024):	USD 30.000,00
3.	After provision of status report 2 (August 2025):	USD 11.787,20
4.	After receipt of the final report (July 2026):	USD 27.946,80
	**Total:**	**USD 139.734,00**

Payments are released according to the payment schedule above on receipt of an invoice from the Principal Investigator’s institution.

An annual update report must be completed on request by ITI Headquarters. Twenty percent of the agreed grant amount will be held back and transferred only after receipt of a complete final report. All reports will be forwarded to the ITI Research Committee.

A copy of the publication resulting from the ITI-funded project must be provided to ITI Headquarters as soon as available.

Any unspent funds after completion of the project should be returned to the ITI.

The ITI Research grant terms and conditions apply as signed in the original grant application.

This project has been registered as ITI Grant number 1744-2023 which must be quoted in all correspondence.

With best regards

Prof. Dr. Dr. Bilal Al-Nawas	Deborah Bader
Chair of the ITI Research Committee	Manager Research

Information on how to issue an invoice:

Invoice must be addressed to:

ITI Foundation
Research Grant Administration
Peter Merian Weg 10
4052 Basel
Switzerland

Invoices must be made out:

- in English or German
- in the currency of the grant award
- on the First Applicant's[1] institution's letterhead and quote the institution's name and address

Invoices must quote:

- the ITI Grant number
- the **First Applicant's institution's VAT identification number**
- the **ITI Foundation's Swiss VAT number: CHE-101.991.665 MWST**
- the name and the address of the bank
- the name of the account holder
- the bank account number and SWIFT (non-European countries)
- IBAN and SWIFT (Europe)

Invoices are to be emailed to ITI Headquarters to research@iti.org

[1]First Applicant = individual listed as «First Applicant" on the ITI Research Grant application form submitted to the ITI
