# Supplementary material for: 3D printing vs traditional workflow for the fabrication of mandibular implant overdentures: study protocol for a mixed-methods cross-over RCT
Source: Trials. 2024 Apr 16;25:267. doi: 10.1186/s13063-024-08097-7 (PMC11022432; doi:10.1186/s13063-024-08097-7)
Supplement: Supplementary file 4 — Additional file 4. Consent form in English. [file 13063_2024_8097_MOESM4_ESM.docx]

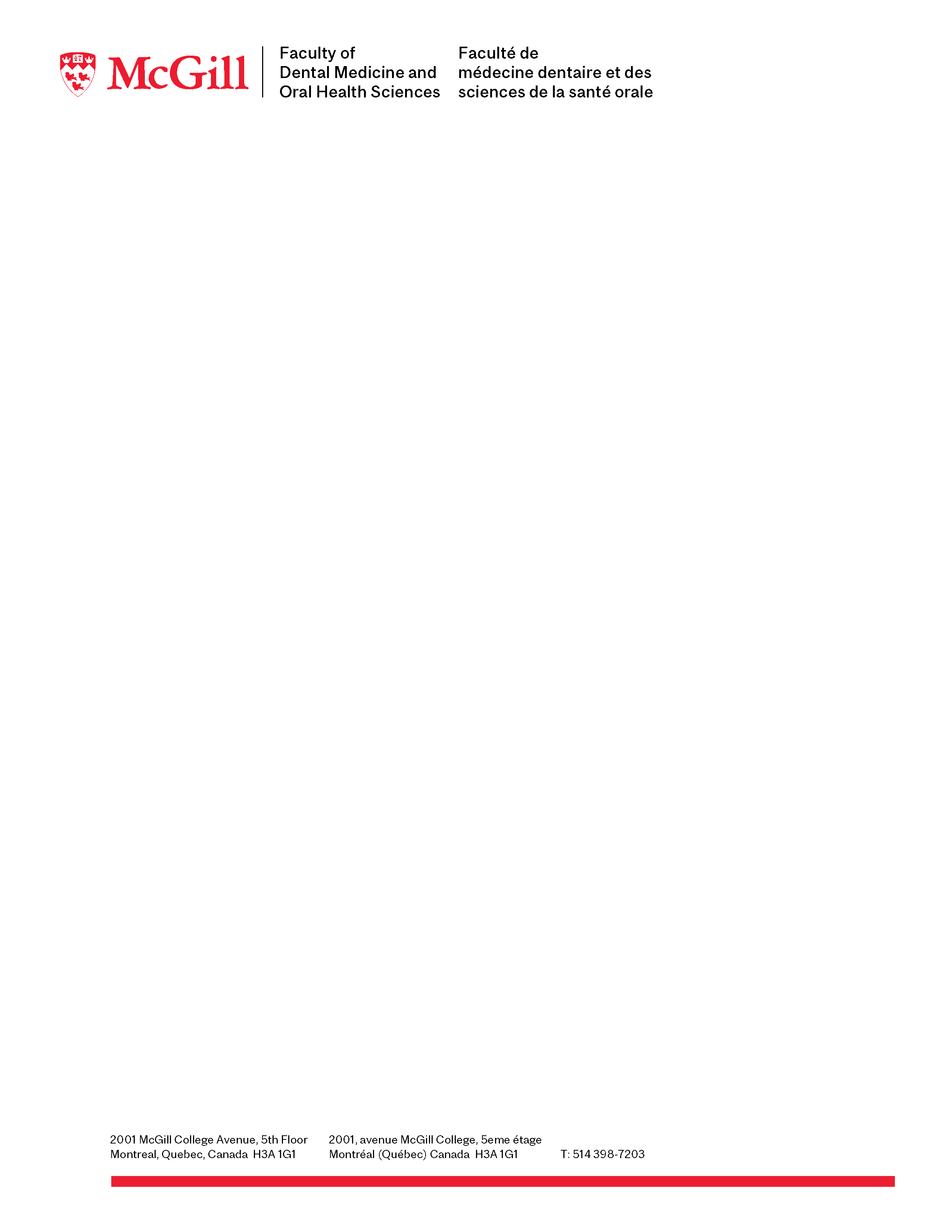


**INFORMATION AND CONSENT FORM**

| **Research Study Title:** | 3D printing vs traditional workflow for the fabrication of implant mandibular overdentures: A randomized cross-over clinical trial |
| --- | --- |
| **Protocol number:** | A00-M29-23B (22-07-089) |
| **Researcher responsible for the research study:** | Dr. Raphael F de Souza  Faculty of Dental Medicine and Oral Health Sciences, McGill University |
| **Co-Investigator(s)/sites:** |  |
|  |  |
| **Sponsor:** | ITI (International Team for Implantology), grant 1744-2023 |

**INTRODUCTION**

We are inviting you to take part in this research study because you have no natural teeth and currently wear full dentures. You should be: (1) 60 years or older; (2) Not have had a tooth extraction within the past 12 months; (3) desire to receive new dentures; (3) two dental implants in the lower jaw; (4) desire to receive both upper and lower dentures; (5) good understanding of spoken English or French; (6) Ability to provide written informed consent.

However, before you accept to take part in this study and sign this information and consent form, please take the time to read, understand and carefully examine the following information. You may also want to discuss this study with your family doctor, a family member or a close friend.

This form may contain words that you do not understand. We invite you to speak to the researcher responsible for this study (the “study doctor”) or to other members of the research team and ask them to explain to you any word or information that is unclear to you before you sign this form.

**BACKGROUND**

Complete tooth loss or edentulism is still a grievous oral health problem in Canada and worldwide. It is more common in older adults and is expected to remain frequent for several decades.

Studies have suggested that complete tooth loss leads to worse general health in older adults and even earlier mortality. Some diseases are more common in toothless individuals, including gastrointestinal cancer, heart disease, dementia and stroke. Tooth loss can also reduce well-being, with depressive symptoms worsening after losing all teeth. Poorer chewing is also common with tooth loss and is closely associated with reduced self-esteem. Besides that, many toothless individuals avoid social activities due to shame when smiling, speaking or eating.

Digital technology can help older adults to receive dentures. For example, dentures can need up to 5 clinical sessions to be ready. With digital methods dentures can be delivered with 2 to 4 sessions. Also, denture patients can have certain steps done by distance, like checking the appearance of their future teeth. Two of the advantages of less sessions are: (1) a lower cost, (2) lower risk to get sicknesses like COVID-19 or the flu. Dentures done with digital methods also tend to fit better in the mouth than traditional dentures.

However, to make sure those advantages make sense for our patients, we need to run clinical studies. This way, this study will compare digital dentures to the traditional ones, regarding your satisfaction and preferences. We will also have a dentist checking their quality and estimate what would be their cost for patients in general. Our results will guide dentists and patients when choosing the most appropriate method for their needs.

**PURPOSE OF THE RESEARCH STUDY**

The purpose of this study is to learn about your satisfaction with a novel computer-aided design and manufacturing (CAD/CAM) technology for providing full denture prostheses comparable to traditional methods, regarding patient-and clinician-perceived quality.

Secondary purposes are to compare if one of the two tested methods incur higher costs from the patient perspective.

For this research study, we will recruit 26 participants, men and women, aged 60 years or more.

**DESCRIPTION OF THE RESEARCH PROCEDURES**

This research study will take place at the following site:

1. Clinical research unit at the Faculty of Dental Medicine and Oral Health Sciences, McGill University, 2001 McGill College Ave, Montreal, Quebec H3A 1G1

**1. Duration and number of visits**

Your participation in this research project will last 8 months and will include 11 visits, besides the screening visit: 5 for denture fabrication, 4 for post-delivery adjustments* and 2 for data collection. Each visit will last around 90 minutes.

**Adjusting dentures may demand you to come for some extra visits, as usual in a standard clinical practice.*

**2. Study devices**

We will recruit toothless participants (any sex/gender) among previous patients of McGill University, based on the following eligibility criteria: mandibular tooth loss, age ≥60 years, need for new mandibular overdentures, ability to complete questionnaires, and previous treatment with implants in the lower jaw.

Each participant will receive two pairs of mandibular overdentures: (i) one with 3D images of the mouth, virtual prosthetic design and 3D printing (CAD/CAM); (ii) a control pair, by traditional clinical and laboratory methods. Each pair will be used for 3 months according to a random sequence (total follow-up: 6 months).

**3. Tests and procedures**

During your participation in this research study, the study doctor or a member of the research team will conduct the following tests and procedures:

| **DESCRIPTION OF STUDY PROCEDURES** | |
| --- | --- |
| **Procedure** | **Description** |
| 1. Screening | - Initial questionnaire, mouth exam *(as the standard of care)*  - Consent form *(only for the study)* |
| 2.Molding, 1^st^ time | - Completing your dental exam forms  - Taking molds of your upper and lower jaws *(primary impressions)*  - Obtaining 3D pictures of your upper and lower jaws *(intraoral scanning)* |
| 3. Molding, 2^nd^ time | - Taking more accurate molds of your mouth *(secondary impressions)* |
| 4. Shaping denture bases | - Verifying provisional denture bases for their fit and shape  - Shaping wax rims on denture bases for the desired position of denture teeth  - Registering the position of your jaws when biting *(occlusal plane, vertical dimension and centric relation)*  - Taking 3D pictures of your face and mouth with bases |
| 5. Denture try-in | - A wax replica of your future denture will be checked in the mouth *(traditional denture try-in)*  - Showing you a 3D photo with the denture teeth *(virtual try-in)* |
| 6. Denture delivery | - Adjusting the fit of new dentures and their teeth  - Hollowing your denture and installing retentive components |
| 7. Denture adjustments | - Adjusting the fit of the dentures and their teeth. You may have some sore spots on the gums before adjustment (normal with new dentures) |
| 8. Data collection | - Questionnaires and mouth exam *(only for the study)* |
| 9. Interview | - You will discuss your feelings and preferences with each denture pair. This will happen in private and out of the clinic *(only for the study)* |

The schedule of procedures for each visit is listed below:

| **SCHEDULE OF STUDY PROCEDURES** | | | | | | | | | | | |
| --- | --- | --- | --- | --- | --- | --- | --- | --- | --- | --- | --- |
| **Procedure** | **Visit (V) 0** | **V 1** | **V 2** | **V 3** | **V 4** | **V 5**  **(Day 0)** | **V 6 & 7** | **V 8**  **(3 months)** | **V 9 & 10** | **Visit 11**  **(6 months)** |  |
| 1. Screening* | **X** |  |  |  |  |  |  |  |  |  |  |
| 2.Molding, 1^st^ time* |  | **X** |  |  |  |  |  |  |  |  |  |
| 3. Molding, 2^nd^ time |  |  | **X** |  |  |  |  |  |  |  |  |
| 4. Shaping denture bases |  |  |  | **X** |  |  |  |  |  |  |  |
| 5. Denture try-in |  |  |  |  | **X** |  |  |  |  |  |  |
| 6. Denture delivery |  |  |  |  |  | **X** |  |  |  |  |  |
| 7. Denture adjustments |  |  |  |  |  |  | **X** |  | **X** |  |  |
| 8. Data collection |  |  |  |  |  | **X** |  | **X** |  | **X** |  |
| 9. Interview |  |  |  |  |  |  |  |  |  | **X** |  |
| **Study site** | **McGill Faculty of Dental Medicine and Oral Health Sciences** | | | | | | | | | | |

* We may take new radiographs (panoramic and/or of the implants) if needed

**PARTICIPANT’S RESPONSIBILITIES**

- As for standard dental treatment, please communicate if you cannot come to any of the scheduled appointments. Plus, please tell us if something changes in your health, even if that may not seem relevant.
- Dentures need regular care; please brush them at least twice/daily. We recommend you to see a dentist regularly after the end of the study, at least once/year as participation in this study does not replace these regular checkups in any way.
- You will be responsible for the costs of dental care after the study ends. Eventual repairs and changes of components will be necessary, as normal for dentures and implants. Fees may vary in different clinics, but you may expect fees of nearly $100 for repairing a component. Other repairs may be more expensive, such as refitting the denture with acrylic ($394 or more, depending on how it is done).

**BENEFITS ASSOCIATED WITH THE RESEARCH STUDY**

You may or may not personally benefit from your participation in this research project. However, we hope that the study results will contribute to the advancement of scientific knowledge in this field and help us find better treatments for patients.

All treatment involved in this study (adapting your denture and changing implant components during the 6-month period) will be done free of charge.

**RISKS ASSOCIATED WITH THE RESEARCH STUDY**

Both study devices are licensed for patient use and sold in Canada and United States. This way, risks associated with treatment are the same expected for minor oral surgery and standard dental implants/dentures.

If you have noticed side effects, whatever they may be, during this research study, you must tell the study doctor immediately, regardless of whether you think these effects are related to the implants or components. Even once your participation in the study is over, do not hesitate to contact the study doctor if you experience a side effect that may be linked to the study devices.

The study doctor and members of his or her team will answer any questions that you may have regarding the risks, discomforts and side effect associated with this study. Also, at each visit, the study doctor and members of his or her team will ask you questions about any side effects you may have experienced.

**Risks associated with dentures**

You may experience some sore spots under your dentures after the placement of retentive components. If this happens, the dentures will be adjusted for you as necessary. Allergic reactions to dental materials (such as the acrylic mixture used to bond components and denture) are rare but might also occur.

Your lower denture might break after we place the implant retentive components. We may have to drill the denture before placing them. This can weaken the denture. If this happens during the study timeline, we will fix/repair it at no cost.

We do not expect risks or complications from the x-rays or other exams. This includes data collection and interviews.

**OTHER POSSIBLE TREATMENTS**

You do not have to take part in this study to receive medical care for your condition. Other options exist such as: (1) new full dentures or repairs (“relinings”); (2) more than two implants in the lower jaw; (3) implants in the upper jaw. We encourage you to discuss with the study doctor all available options.

**VOLUNTARY PARTICIPATION AND THE RIGHT TO WITHDRAW**

Your participation in this research project is voluntary. Therefore, you may refuse to participate. You may also withdraw from the project at any time, without giving any reason, by informing the study doctor or a member of the research team.

Your decision not to participate in the study, or to withdraw from it, will have no impact on the quality of care and services to which you are otherwise entitled, or on your relationship with the study doctor or clinical team.

The study doctor, the Research Ethics Board, the funding agency, or the Sponsor may put an end to your participation without your consent. This may happen if new findings or information indicate that participation is no longer in your interest, if you do not follow study instructions, or if there are administrative reasons to terminate the project.

However, for safety and future result analyses, before you withdraw from the study we ask you to notify the contact person in the research team, verbally or in writing.

If you withdraw or are withdrawn from the study, the information and biological material already collected for the study will be stored, analyzed and used to ensure the integrity of the study.

Any new findings that could influence your decision to stay in the research project will be shared with you as soon as possible.

**CONFIDENTIALITY AND PRIVACY**

During your participation in this study, the study doctor and their team will collect and record information about you in a study file. They will only collect information required to meet the scientific goals of the study. You will not be asked for personal or sensitive information in a public setting. Instead, information will be collected in a private space where the discussion cannot be observed or overheard by others. Investigators will also limit the information collected to the information that is essential for research purposes, and only once informed consent has been obtained from you.

The study file may include information from your medical chart, including your identity, concerning your past and present state of health, your lifestyle, as well as the results of the tests, exams, and procedures that you will undergo during this research project. Your research file could also contain other information, such as your name, sex, date of birth and ethnic origin.

The research forms and x-rays will be sent to Dr. de Souza’s office and stored for 25 years for the exclusive objectives of this study and then destroyed. His office is located at McGill University, Strathcona Anatomy & Dentistry Building, 3640, University St., room M/65A, Montreal (QC) H3A 2B2.

The collected data will be only stored in the OneDrive cloud server of the principal doctor, the internal drive of the professional computer of the principal doctor, and the external hard drive of the principal doctor. These will be kept secure by a password to which only the principal doctor will have access.

For the purpose of any publications, your demographic information might be shared in the paper without any identification of you. Upon request of the sponsor, your anonymized data might be shared with them. The data will be stored anonymously, pertaining to your specific coded ID. That being said, you cannot be identified through the shared data.

All the information collected during the research project will remain strictly confidential to the extent provided by law. You will only be identified by a code number. The key to the code linking your name to your study file will be kept by the study doctor only.

To ensure your safety, a copy of this information and consent form (including the type of implant/components in use and x-ray results) will be placed in your medical chart. As a result, any person or company to whom you give access to your medical chart will have access to this information.

The study doctor might forward your coded data to the sponsor or their representatives upon their request.

The Sponsor may share the coded study data with their commercial partners. However, the sponsor and any international partners will respect the confidentiality rules in effect in Quebec and Canada, regardless of the country to which your data may be transferred.

The study data will be stored for 25 years by the principal investigator (Dr de Souza).

For depositing research data, we might deposit anonymized datasets in the McGill Dataverse repository. According to McGill, all data are stored securely on servers located in Canada. The anonymized data may be published or shared during scientific meetings; however, it will not be possible to identify the participants.

For monitoring, control, safety, and security, your study file as well as your medical charts may be examined by a person mandated by Canadian or international regulatory authorities, such as Health Canada, as well as by representatives of the study sponsor, the institution, or the Research Ethics Board. All these individuals and organizations adhere to policies on confidentiality.

You have the right to consult your study file in order to verify the information gathered, and to have it corrected if necessary.

However, in order to protect the scientific integrity of the research project, accessing certain information before the project is ended may require that you be withdrawn from the study.

**INCIDENTAL FINDINGS**

Material incidental findings are findings made in the course of the study that may have significant impacts on your current or future wellbeing or that of your family members. A material incidental finding concerning you in the course of this research will be communicated to you and to a health professional of your choice.

We will examine you according to standard practices in dentistry during “visit 1”. This may reveal certain diseases that are outside our study goals but have importance for your well-being. Examples are: (1) infection of your mouth; (2) tumours or cysts only visible by x-ray. We will tell you about such a finding and refer you to adequate treatment when needed. This will happen regardless of your inclusion or not in this study. If we have any incidental finding during any visit, we will do the same.

**MARKETING POSSIBILITIES**

The research results, including those following your participation in this study, could lead to the creation of commercial products. However, you will not receive any financial benefits.

**FUNDING OF THE RESEARCH PROJECT**

The study doctor and the institution have received funding from the sponsor for the completion of the research project.

**COMPENSATION**

You will receive an amount of 25$ per study visits 8 and 11, for a total of 02 visits, for a total amount of 50$ for costs and inconveniences incurred during this research study. If you withdraw from the study, or are withdrawn before it is completed, you will receive compensation proportional to the number of visits you have completed. Denture repairs and replacement of implant attachment components will be offered to you for free for the duration of this research study.

**SHOULD YOU SUFFER ANY HARM**

Should you suffer harm of any kind following administration of the study drug, or following any other procedure related to the research study, you will receive the appropriate care and services required by your state of health.

By agreeing to participate in this research project, you are not waiving any of your legal rights nor discharging the study doctor, the sponsor or the institution, of their civil and professional responsibilities.

**CLINICAL TRIAL REGISTRATION**

A description of this clinical trial is available on <http://www.ClinicalTrials.gov> (project NCT06155630). This Website will not include information that can identify you. At most, the Website will include a summary of the results. You can search this Website at any moment, or access <https://classic.clinicaltrials.gov/ct2/show/NCT06155630> directly.

**CONTACT INFORMATION**

If you have questions or if you have a problem you think may be related to your participation in this research study, or if you would like to withdraw, you may communicate with the study doctor or with someone on the research team at the following number: Dr. Raphael de Souza (principal investigator) at McGill University, telephone: (514) 913-7174; email: [raphael.desouza@mcgill.ca](mailto:raphael.desouza@mcgill.ca).

For any question concerning your rights as a research participant taking part in this study, or if you have comments, or wish to file a complaint, you may communicate with:

(1) the Research Ethics Officer at McGill University (Mrs. Ilde Lepore) by email: ilde.lepore@mcgill.ca or by telephone at (514) 398-8302.

**OVERVIEW OF ETHICAL ASPECTS OF THE RESEARCH**

The McGill University Research Ethics Board reviewed this study and is responsible for monitoring it at all participating institutions in the health and social services network in Quebec.

| **Research Study Title:** | 3D printing vs traditional workflow for the fabrication of implant mandibular overdentures: A randomized cross-over clinical trial |
| --- | --- |

**SIGNATURES**

***Signature of the participant***

I have reviewed the information and consent form. Both the research study and the information and consent form were explained to me. My questions were answered, and I was given sufficient time to make a decision. After reflection, I consent to participate in this research study in accordance with the conditions stated above.

I authorize the research study team to have access to my medical record for the purposes of this study.

- I authorize the doctor in charge of this research study to communicate with me directly to ask if I am interested in participating in other research:

Yes

No

- I authorize the study doctor to inform my treating physician that I am taking part in this study:

Yes  Name and contact information of treating physician: ___________________________

No

I do not have a treating physician/I am no longer being followed by my treating physician

- I agree that my anonymized data be shared with the study sponsor and other commercial partners and may be made available in a data repository:

Yes

No

I understand that the study doctor will send my treating physician health information if it will be useful for my care.

Name of participant Signature Date

***Signature of the person obtaining consent***

I have explained the research study and the terms of this information and consent form to the research participant, and I answered all his/her questions.

Name of the person obtaining consent Signature Date

***Commitment of the principal investigator***

I certify that this information and consent form were explained to the research participant, and that the questions the participant had were answered.

I undertake, together with the research team, to respect what was agreed upon in the information and consent form, and to give a signed and dated copy of this form to the research participant.

Name of the principal investigator Signature Date
