# Supplementary material for: 3D printing vs traditional workflow for the fabrication of mandibular implant overdentures: study protocol for a mixed-methods cross-over RCT
Source: Trials. 2024 Apr 16;25:267. doi: 10.1186/s13063-024-08097-7 (PMC11022432; doi:10.1186/s13063-024-08097-7)
Supplement: Supplementary file 3 — Additional file 3. Ethical approval document. [file 13063_2024_8097_MOESM3_ESM.pdf]

McGill | Faculty of Medicine and Health Sciences | Faculté de médecine et des sciences de la santé

3655 Sir William Osler #633
Montreal, Quebec H3G 1Y6

3655, Promenade Sir William Osler #633
Montréal (Québec) H3G 1Y6

Tél/Tel: (514) 398-3124

September 21, 2023

Dr. Raphael F. de Souza
Faculty of Dental Medicine and Oral Health Sciences
2001, ave McGill College, Suite 534
Montreal, QC H3A 1G1

RE: **A00-M29-23B (22-07-089)**
3D printing vs traditional workflow for the fabrication of implant mandibular overdentures: A randomized cross-over clinical trial

Dear Dr. de Souza,

On September 11, 2023, at a meeting of the Institutional Review Board, the above-referenced re-submitted study received a full Board review.

The Committee identified the following concerns for your response.

Study Protocol

a) Science of the study is acceptable.

Consent form

b) Consent form needs to be on letterhead.
c) Please clarify if data is coded or anonymized. If the data is to be kept for 25 years, it needs to be anonymized.

The study was approved for a period of 12 months, pending the submission of an appropriate response and revisions to the above concerns.

Please send all correspondence to submit2irb.med@mcgill.ca.

Kind regards,

Roberta M. Palmour

Roberta Palmour, PhD
Chair
Institutional Review Board

cc: A00-M29-23B (22-07-089)
